# The Electro-Deposit Plates

**Published:** 1892-01

**Authors:** 


### CORRESPONDENCE.

### The Electro Deposit Plates.

DEAR EDITOR:—

Please allow me a little space to reply to a report on Dental Art and Invention of the Illinois State Dental Society, in regard to the above plates. The complaint is made that they discolor, and that one gentleman writes, "that it is very embarrassing to put into the mouth a *gold* plate and have it return in a few weeks looking like pot-metal." These plates are not gold plates. They are not and never have been sold as gold plates, but as *silver* plates, covered with gold, and the dentist should always state this fact to his patients. They are liable to tarnish and the patient should be so informed, and that it is necessary to keep them well cleansed.

Mr. Joseph G. Ward, the patentee of this process, wears a full upper and lower plate, and I saw them a few days ago, and after three years wear they are as bright and beautiful as when first made. Mr. Ward cleans them once a day with a soft brush and hand sapolio.

Then, too, many of the dentists never return their plates for the final finish of gold. Out of the 1,400 plates made, fully one-third have never been returned for the finishing coat. As only enough gold is put on the first time to vulcanize over, and the silver surface is more or less exposed in finishing up, it is not surprising they tarnish.

Some plates are returned to be recoated because they are discolored. Some of these plates look as if they had never made the acquaintance of a brush. Is it a wonder then, they tarnish? I have seen rubber plates badly discolored from the same cause. I have seen pure gold fillings, in some mouths, black; a little cleaning restores them. So give the deposit plate a fair show.

I have used, in my own practice, some sixty of these plates, and they give me and my patients entire satisfaction. I always have from one to two extra dwts. of gold put on.

Experiments are being made with a view to remedy the tendency to discoloration, and it is hoped, will be successful, and if so, I can confidently say it is the best dental plate made, for the success of a denture depends largely on its accuracy of fit, and a deposit plate must fit, if the model is correct; it can not do otherwise. The strength of the deposit plate is equal to 18k. rolled gold plate. Any can satisfy themselves of this by taking a piece of each and bending back and forwards until it breaks.

It is as strong as gold; it is better than rubber; its fit is better than any other plate. The only thing against it is that in some mouths it is liable to discolor, and this slight defect can be overcome by extra gold and *cleanliness*.

Respectfully,

C. S. Stockton,

November 1, 1891. 17 W. Park St., Newark, N. J.

FOR DENTAL REGISTER.

*Dr. Taft*:—

For more than three years I have been using a Shaw Dental Engine, and like it very much but for the bother of elbow springs breaking. After using about two dozen duplex springs I procured a single spring manufactured by Hood & Reynolds, of Boston, that is sold at ten cents. This I covered with a piece of French rubber nursing bottle tubing, the same length as spring which makes a good sleeve. I have put this spring to as severe tests as any elbow spring that I ever had and it has stood me over a year. Some of my friends have used the same for several months and report equal success.

We can recommend this to any who have been annoyed by broken elbow springs as worthy of a trial.

C. W. STAPLES, D.D.S.

